# Appendicitis from the Standpoint of the Physician

**Published:** 1904-09

**Authors:** 


### APPENDICITIS FROM THE STANDPOINT OF THE PHYSICIAN.

S. G. Bonney, Denver (*Jour. A. M. A.*, July 9, 1904) calls attention:

First, to the supreme importance of early diagnosis, which, if obtained at once, affords a key for the satisfactory solution of the much-disputed problem as to the time of operation.

Second, in cases of longer duration, to the necessity of a most careful and detailed review of the previous manifestations, with a deliberate and well-considered differentiation of the several phases, to the end that a non-vacillating purpose may be evolved, subject only to the influence of such clinical developments as may obtain through intelligent observation.

It is insistently urged that a painstaking physical examination of the abdomen should be made in all cases of abdominal pain, regardless of the possible slight degree of severity of the pain, the presence or absence of chill, fever, nausea or vomiting. Not infrequently is a sharply localized area of resistance and tenderness found over the region of the appendix, without the other oft-associated symptoms of the disease. A diagnosis rendered possible at this time by a careful examination is of transcendent importance.

It is furthermore contended that the absence of definite physical signs in the right iliac fossa in the presence of sudden chill, fever, nausea, vomiting, acute general abdominal pain and prostration offers no reliable assurance of the non-existence of a most serious involvement of the appendix.

The contention is made that there is no constant definite relation between the severity of the symptoms, together with the abdominal signs, and the degree or nature of the actual pathologic change within the abdomen. There is no invariable rule by which to hazard an opinion on the existence of gangrene, perforation, abscess, circumscribing adhesions or localized peritoneal inflammation. The pathologic condition, without opening the abdomen, is at best a mere matter of conjecture even during convalescence or in the apparent absence of unfavorable symptoms.

It is obvious in the course of an acute appendical attack that the feeling of security engendered by the absence of distinctly unfavorable or alarming symptoms is often a most unsafe delusion. It goes without saying that no one can see into the abdominal cavity and describe the pathologic process in the neighborhood of the appendix, or estimate with accuracy the imminence of threatening morbid change. One man's dictum is about as authoritative and worthy of credence as another's, other things being equal.

It is conceded that there are certain times and conditions when it is perhaps more conservative to delay operative interference, notably when the case is not seen until after the lapse of over thirty-six hours, and when at the same time there is an apparent remission of the acute symptoms and a more or less amelioration of the physical signs. Reference is here made to the diminution of pain, lowering of pulse and temperature, absence of vomiting or chill, lessening of distension, and perhaps passage of gas or fecal matter. At such a time it is fair to assume, provisionally, that adhesions are forming and that the inflamed area is being effectually walled off from the general peritoneal cavity, or that the inflammation itself has not as yet penetrated the peritoneal covering. In the absence of a return of unfavorable symptoms, it should be recognized that there is perhaps less to gain and more to lose by a meddlesome interference at this particular period. To say the least, however, this time of observation, even at best, is one of anxious responsibility. Pending a recurrence of the previous acute symptoms or the development of other manifestations indicating an extension of the process, it is justifiable to refrain from surgical interference. Immediately, however, on the recurrence of pronounced chill or vomiting, with increased elevation of temperature or renewed abdominal pain, the indications for operation are imperative and brook no delay.

It is well to remember that in the event of an acute appendicitis, definitely and legitimately demanding an operation on its merits, few complicating conditions should furnish a contraindication. In such an emergency the consideration pertains solely to the choice of the lesser of two evils, and the mere existence of tuberculosis, kidney or heart lesions often affords insufficient grounds for hesitation or delay.

In all cases when operation is advocated it should be made clear, not that the operation is absolutely necessary for recovery, but that the chances for recovery are greater with, than without it. The existence of leucocytosis as offering indications for the necessity of operation is of comparatively minor importance.

In doubtful cases it must be emphasized that the dangers of operation *per se* are practically nil, and that the dangers of delay are terrible.

